# Gastrointestinal bleeding caused by metastatic testicular choriocarcinoma: a case report and literature review

**DOI:** 10.1186/s12957-022-02670-7

**Published:** 2022-06-16

**Authors:** Pengliang Zhang, Yu Wang, Lishou Xiong

**Affiliations:** Department of Gastroenterology and Hepatology, First Affiliated Hospital of Sun Yat-sen University, Guangzhou, China

**Keywords:** Testicular choriocarcinoma, Metastatic, Gastrointestinal bleeding, Genital examination

## Abstract

**Background:**

Testicular tumor is one of the common solid tumors in young men. Testicular choriocarcinoma is a non-spermatogonial germ cell tumor, which is the rarest of all testicular cancers. Choriocarcinoma usually shows bleeding at the metastatic site, while gastrointestinal involvement is rare.

**Methods:**

Here, we report a case of testicular choriocarcinoma with gastrointestinal bleeding as the first diagnosis and summarize the similar cases all over the world in recent 20 years.

**Results:**

A 28-year-old male was treated with repeated melena for 2 months. No bleeding foci of the stomach, duodenum, colon, and rectum were found in endoscopy, and no bleeding foci of digestive tract was found in selective angiography, but a space occupying lesions of the lung, liver, and upper jejunum were found in chest and abdominal CT. Considering the possibility of a metastatic tumor and the ineffectiveness of medical treatment, the patient was converted to surgical treatment. The postoperative pathology was consistent with testicular choriocarcinoma. The patient received a chemotherapy regimen of paclitaxel, ifosfamide, and cisplatin. At present, the chemotherapy regimen is well tolerated.

**Conclusions:**

The case report confirmed that even if we cannot find the logical relationship between clinical manifestations and genital examination, genital examination should also be part of the patient’s systematic examination.

## Introduction

Gastrointestinal bleeding has many clinical manifestations, including hematemesis, melena, and hematochezia, and its common causes include peptic ulcer bleeding, esophageal and gastric variceal bleeding, gastrointestinal primary tumor bleeding, and biliary tract bleeding, but bleeding caused by extragastrointestinal tumor metastasis to the digestive tract is very rare in clinic. Especially, bleeding caused by testicular choriocarcinoma metastasis to the gastrointestinal tract is more rare. In 1983, Teryn et al. [1] described the first case of jejunal bleeding caused by metastatic testicular choriocarcinoma. This paper summarizes clinical data of a patient with metastatic choriocarcinoma with gastrointestinal bleeding as the clinical manifestation in the First Affiliated Hospital of Sun Yat-sen University, combined with the review of relevant literature in the past 20 years, so as to improve the diagnostic rate of the etiology of gastrointestinal bleeding and reduce misdiagnosis and missed diagnosis.

## Case presentation

### Clinical data

**Fig. 1:**
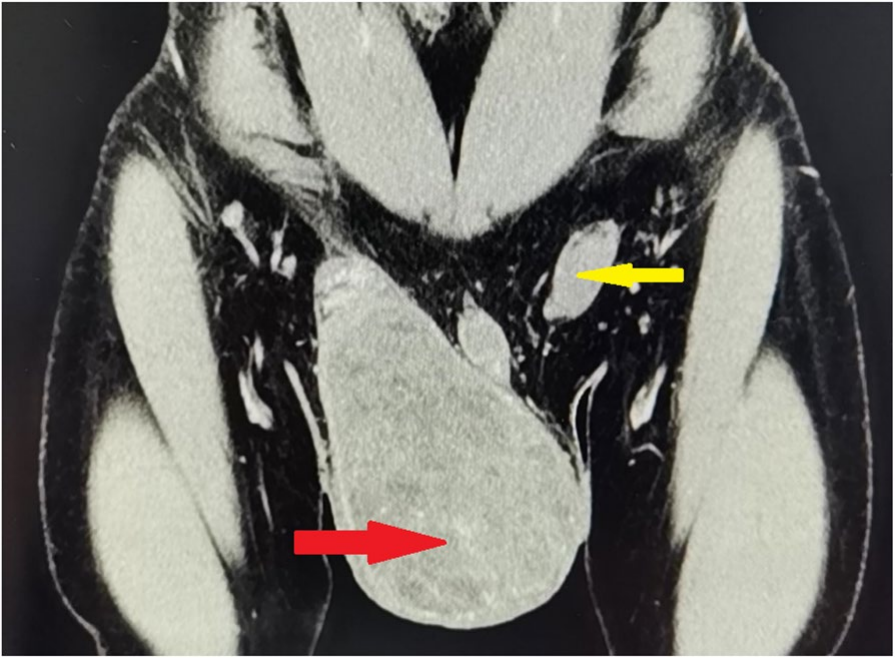
CT images of patients. The red arrow indicates the right testicular tumor, and the yellow arrow indicates the left cryptorchidism

A 28-year-old male patient was admitted to the hospital mainly because of “repeated black defecation for more than 2 months.” He denied the history of peptic ulcers, liver cirrhosis, and portal hypertension. One year ago, he underwent a CT examination of his chest, upper, and lower abdomen and basin due to “right testicular swelling.” The results showed that there was a huge tumor on the right testicle with a clear boundary and protruding into the inguinal canal, about 91mm×86mm×119 mm in size (Fig. 1), uneven density, and medium enhancement in enhanced scanning. The left testicle is located in the inguinal canal. Imaging diagnosis: right testicular mass, considering the possibility of seminoma; Left cryptorchidism: No obvious lesions were found in the chest and abdomen. Improved the examination of tumor markers are as follows: AFP 86.57ng/ml and HCG 11439 mIU/ml. “Radical resection of the right testis” was performed. The postoperative pathology showed that some tumor cells in the testicular tissue were nest-like, adenoid or sieve reticular distribution, rich cytoplasm, large nucleus, and obvious nucleolus. Syncytial cells were seen with massive bleeding. In addition, squamous epithelium, glandular epithelium, cartilage, and small glandular tubular structure can be seen. The morphology is consistent with a malignant mixed germ cell tumor. It is suggested to add immunohistochemical detection to assist in the diagnosis. However, the patient did not undergo immunohistochemical detection and did not undergo any follow-up treatment such as chemotherapy or radiotherapy. Physical examination after admission are as follows: anenergia, severe anemia, pale eyelid conjunctiva, lips and nail bed, and no obvious abnormalities in physical examination such as cardiopulmonary examination. Improved relevant laboratory tests after admission are as follows: HB 3.8g/dl and normal cell anemia. Albumin, liver, and kidney function and coagulation function were normal.

### Diagnosis and treatment process

**Fig. 2:**
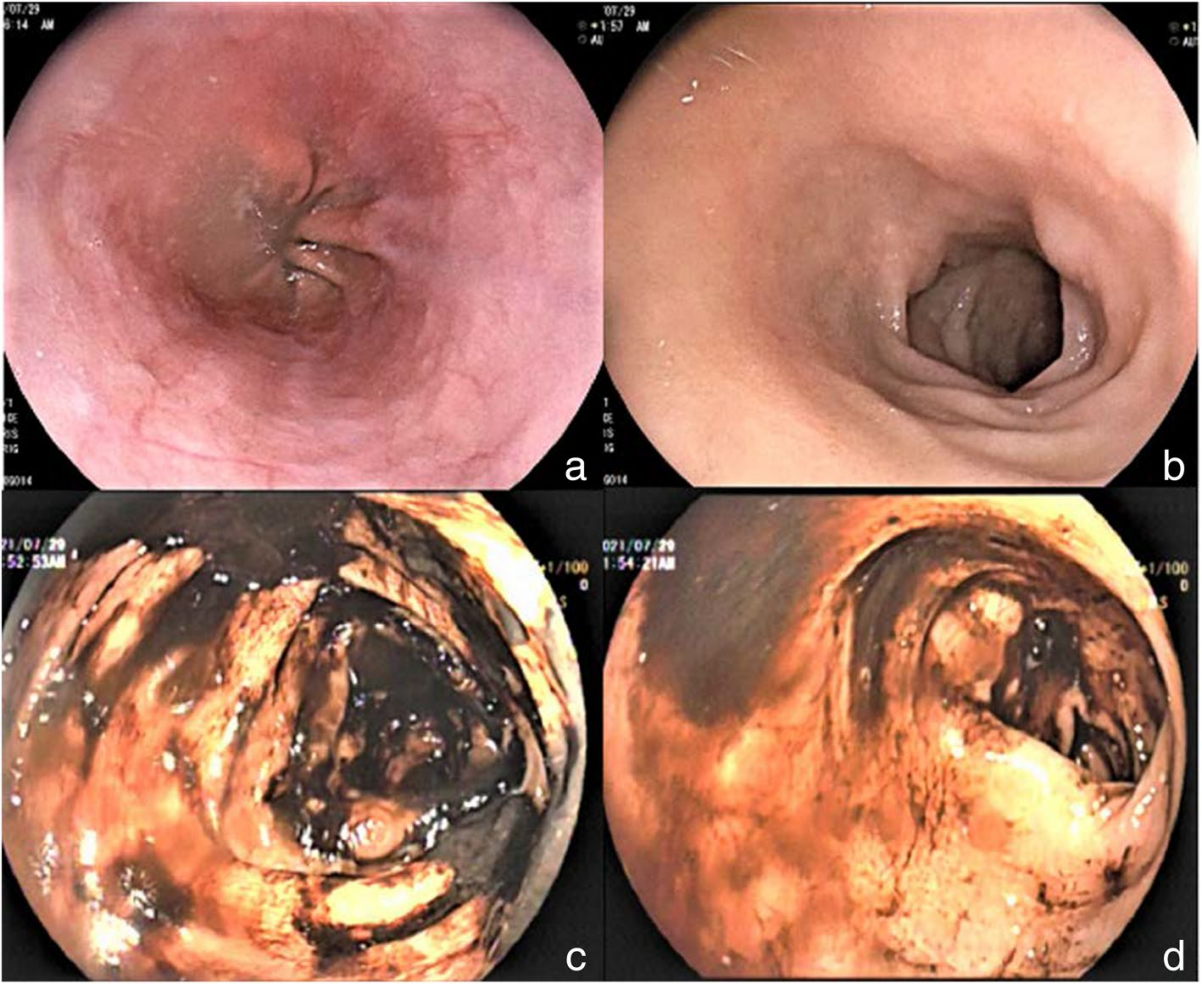
The pictures **a** and **b** indicate gastroscopy results, the pictures **c** and **d** indicate colonoscopy results, and no active bleeding focus is found

**Fig. 3:**
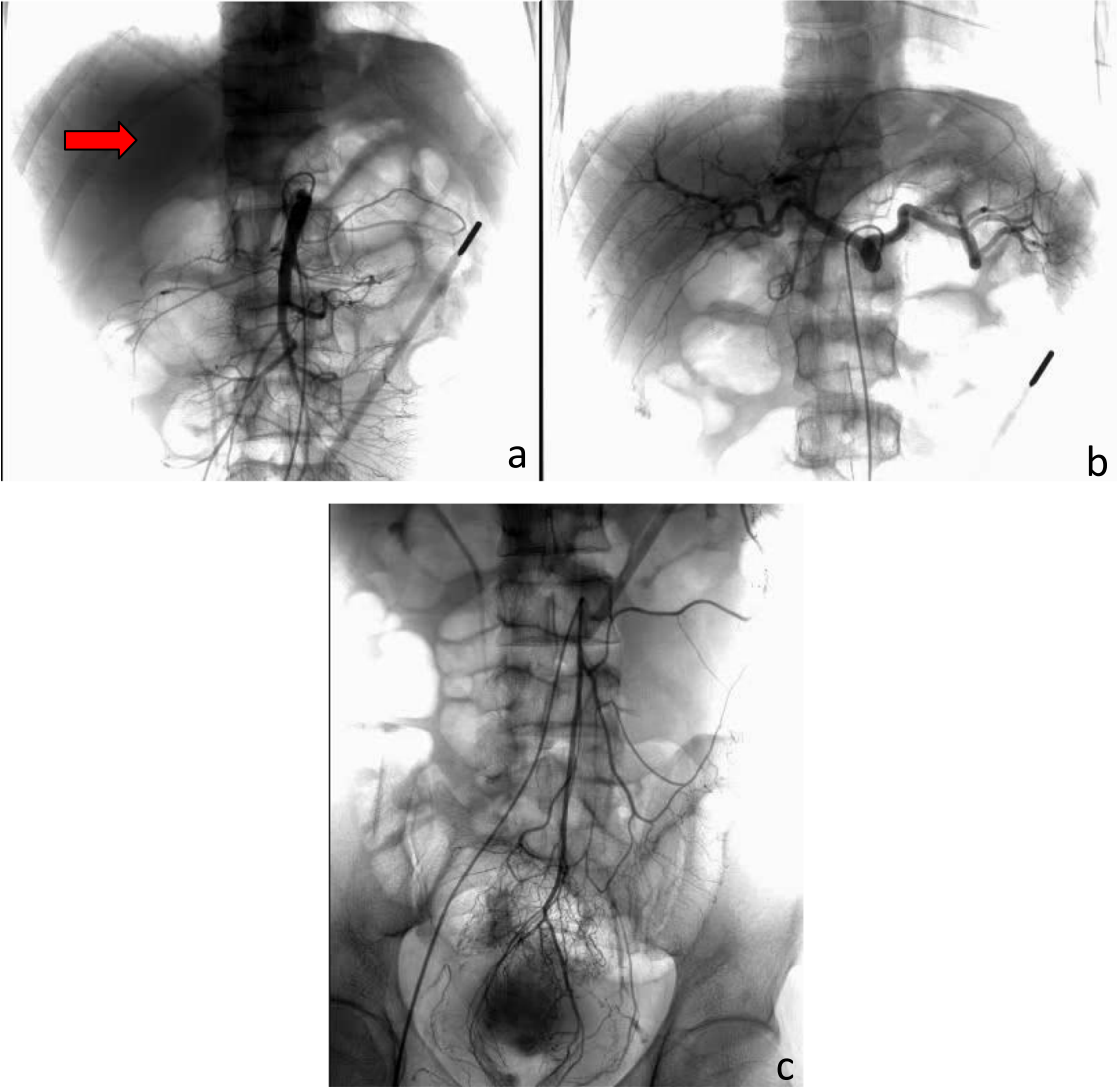
The picture **a** represents the celiac trunk angiography. The red arrow indicates multiple tumor staining in the liver. The picture **b** represents the superior mesenteric arteriography. The picture **c** represents the inferior mesenteric arteriography

**Fig. 4:**
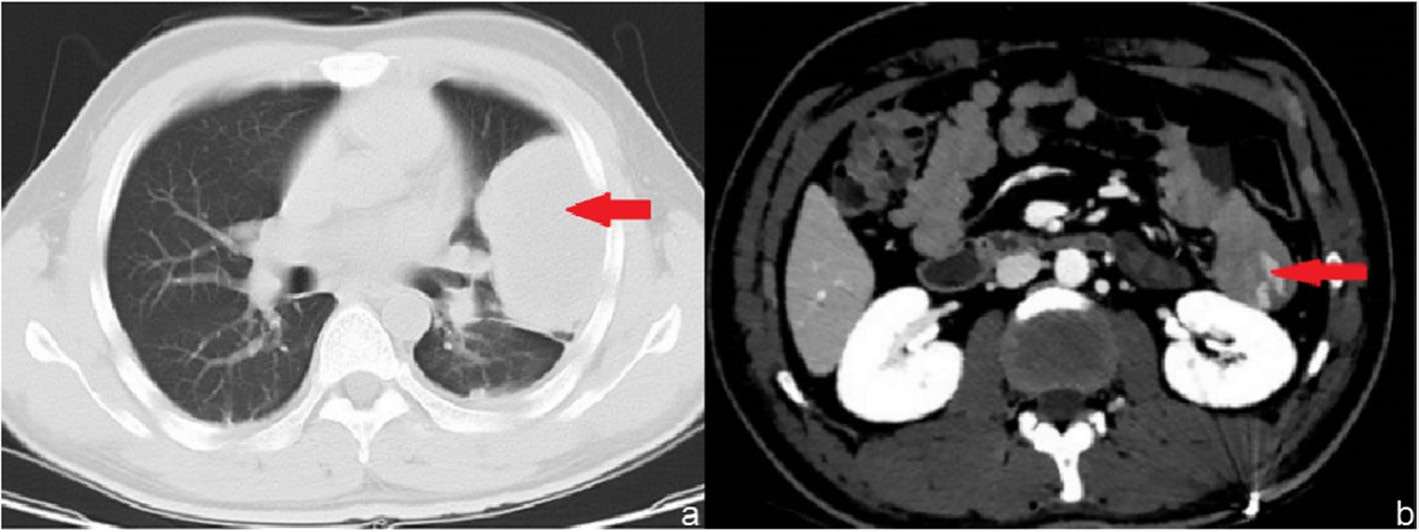
The picture **a** represents the chest CT results, the picture **b** represents the abdominal CT results, and red arrows represent the metastases

After admission, the gastroscopy was urgently improved. Gastroscopy showed that there was no bleeding focus in the stomach and duodenum. Colonoscopy showed that a large number of black feces and fecal water were seen in the intestinal cavity, no bright red blood was found, and no bleeding focus was found in the terminal ileum, ileocecal valve, cecum, appendix opening, colon, and rectal mucosa (Fig. 2). For improved selective angiography, see intrahepatic multiple tumor staining, not excluding the possibility of multiple liver metastases, but no clear gastrointestinal bleeding focus has been found (Fig. 3). Perfect CT examination of the chest and whole abdomen showed that there was a mass in the tongue segment of the upper lobe of the left lung, considering metastasis, and a nodule in the anterior basal segment of the lower lobe of the right lung, considering the possibility of metastasis. Abnormal enhancement of the local intestinal tract in the upper jejunum did not rule out angiogenic lesions (Fig. 4). After medical treatment, the symptoms of melena were relieved and the hemoglobin increased to 8.7g/dl.

**Fig. 5:**
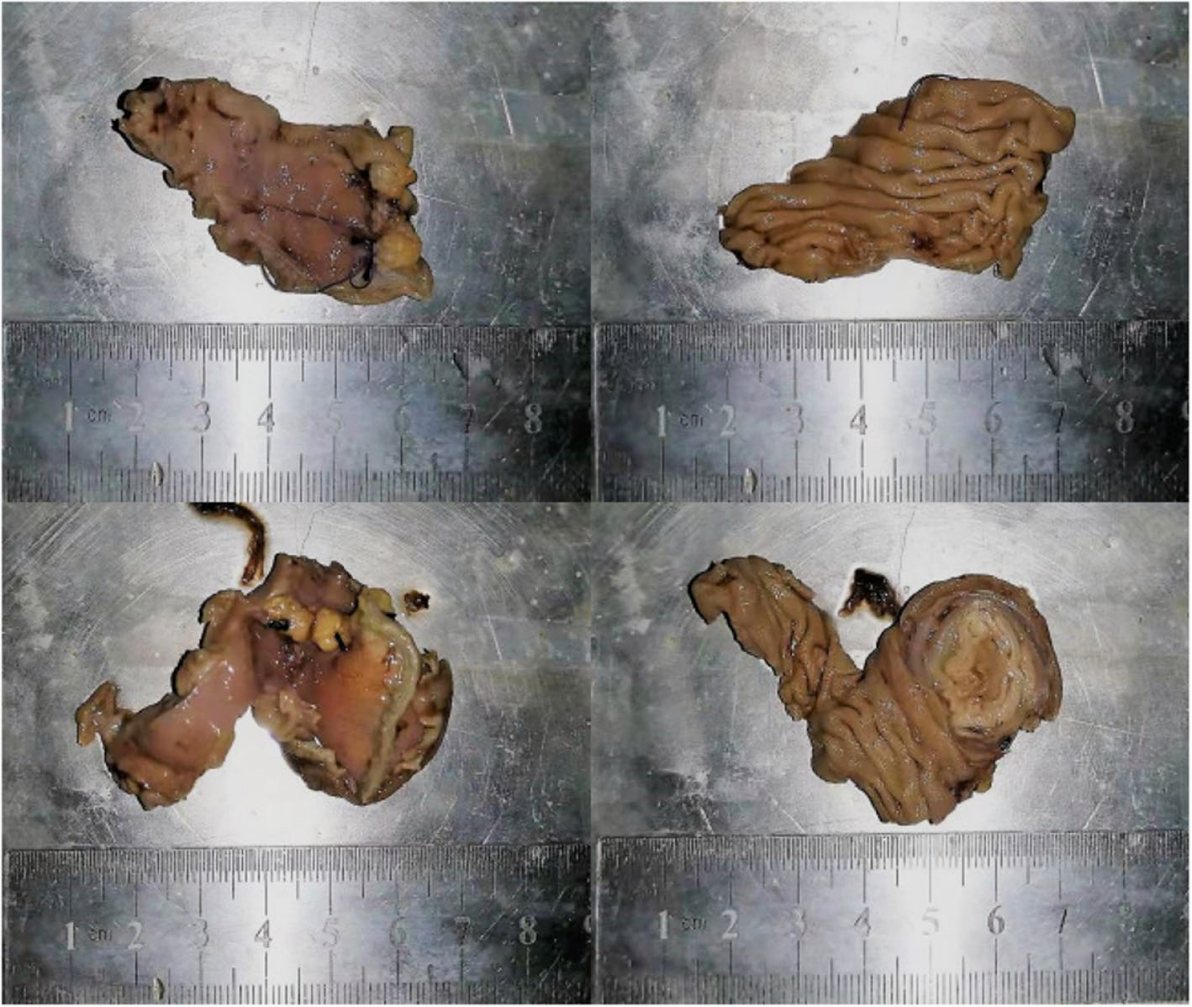
A gross specimen removed surgically (specimen fixed by fixing liquid)

**Fig. 6:**
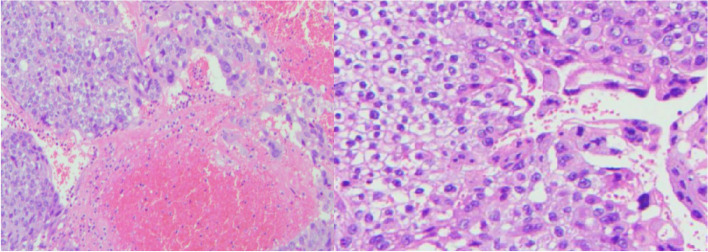
The tumor cells are heterotypic, with mitotic images and focal necrosis

The patient began to eat cold fluid on the 5th day after stopping defecation, but the symptoms of defecation occurred again on the 3rd day. The volume was about 1000ml, the blood pressure decreased to 69/44mmhg, and the hemoglobin was about 5.9g/dl. So on the 10th day after admission, the patient underwent exploratory laparotomy that showed about 15-cm away from treiz ligament, the upper end of jejunum can be overlapped, and soft masses can be touched. The lack intestinal fluid accumulates in this segment of the intestinal cavity. Combined with preoperative CT images, it is considered that the masses here are bleeding sites. Resection of jejunal tumor: the mesentery of the small intestine supplying the tumor segment was ligated and cutoff during the operation, with length of about 5cm (Fig. 5). There was no gastroenterostomy or reconstruction during the operation. The total duration of the operation was 120 min, and the intraoperative bleeding was 100ml. The patient did not enter the ICU for treatment after the operation. The postoperative pathology showed that (small intestine) there was tumor infiltration in the intestinal septum with massive bleeding. The tumor cells showed two forms: one was a rich and bright cytoplasm with a clear boundary, and the other was a multinucleated syncytial cell like. The tumor cells are heterotypic, with mitotic images and focal necrosis (Fig. 6). Immunohistochemistry showed cancer cells CK7 (+), HCG diffuse (+), AFP weak (+), p57 part (+), p53 part (+), CD17 individual cells (+), PLAP focal (+), CD56 individual cells (+), CK20 (-), CDX-2 (-), SATB (-), TTF-1 (-), napsina (-), p40 (-), Sall4 (-), OCT3 / 4 (-), CD30 (-), and syn (-). It is considered to be intestinal metastasis of malignant germ cell tumor, and the metastatic component is mainly choriocarcinoma. The patient stopped bleeding after the operation and is currently receiving further chemotherapy. The chemotherapy regimen is tip (paclitaxel, ifosfamide, and cisplatin). At present, the patient tolerated the chemotherapy regimen well and was discharged for outpatient follow-up. During the 1-month follow-up, the patient showed no signs of recurrent gastrointestinal bleeding.

## Discussion

Testicular cancer is the most common tumor in men aged 15 to 44 [2, 3], which is generally divided into germ cell tumors and non-germ cell tumors. Germ cell tumors include several cell types, roughly divided into seminoma and non-seminoma. Among them, choriocarcinoma is a non-seminoma germ cell tumor, which is the rarest, accounting for 1–3% of all testicular tumors [4]. At present, the cause of testicular choriocarcinoma is not clear, which may be related to a variety of risk factors. Cryptorchidism may be one of the important factors leading to testicular choriocarcinoma. It is reported that the probability of cryptorchidism patients with choriocarcinoma is 20–40 times higher than that of the normal testis [5] in this paper. The patient is left cryptorchidism, which may be one of the important factors leading to testicular choriocarcinoma.

Choriocarcinoma mainly metastasizes through the blood. Because of its strong invasiveness to the blood vessels and tissues, it leads to tissue bleeding and necrosis. Metastasis occurs early and widely. Therefore, when choriocarcinoma is diagnosed, a large number of cases have metastasized, so that most of the initial manifestations are metastasis-related [6]. Because the most common metastatic sites are the lung, liver, and brain [6], patients usually show seizures, stroke-like symptoms, blurred consciousness, and/or hemoptysis. Gastrointestinal metastasis of choriocarcinoma is very rare. Gastrointestinal metastasis occurs in 5% of germ cell tumors. Gastrointestinal metastasis is considered to be the result of direct diffusion or hematogenous diffusion from adjacent retroperitoneal lymph nodes, and direct infiltration is more common than hematogenous diffusion. The small intestine, the most common duodenum, is the most common metastatic site (72%), followed by the esophagus, stomach, and colon [7, 8]. The involvement of the small intestine is characterized by a intestinal obstruction or gastrointestinal bleeding, usually abdominal pain, melena, or anemia. In this paper, the patient was treated with repeated melena as the first symptom, but in the follow-up examination, it was found that there were tumor metastasis in the lung and liver in addition to small intestinal metastasis, but did not show clinical symptoms.

Sixteen cases reported in other journals in the past 20 years were reviewed and analyzed (Table 1). We found that the age of the patients ranged from 17 to 60 years old. Most of them had melena as the first diagnosis symptom (12/16, 75%), and a few had anemia as the first diagnosis symptom (3/16, 18.75%). Only one patient had hematemesis as the first diagnosis symptom, which may be related to the fact that most of the bleeding sites were in the small intestine (10/16, 62.5%), of which the duodenal bleeding was the most, with 6 cases. There were only 4 patients with gastric bleeding alone, and 2 patients with bleeding in the stomach and colon.

**Table 1:** Clinical data of gastrointestinal bleeding caused by metastatic testicular choriocarcinoma

Case	Year	Age	Symptom	Position of bleeding	Treatment	Ending
1 [9]	2002	60	Anemia	Jejunum	Surgical operation	Death
2 [10]	2004	28	Haematemesis	Stomach	Chemotherapy	Death
3 [11]	2004	37	Anemia	Stomach and colon	Chemotherapy	Death
4 [12]	2005	51	Melena	Stomach and colon	Surgical operation+chemotherapy	Death
5 [7]	2009	24	Melena	Intestinum tenue	Surgical operation+chemotherapy	Live
6 [13]	2010	17	Melena	Duodenum	Chemotherapy	Live
7 [14]	2011	25	Melena	Stomach	-	Death
8 [15]	2012	24	Melena	Duodenum	Chemotherapy	Death
9 [16]	2013	24	Melena	Duodenum	Chemotherapy	Live
10 [17]	2013	20	Anemia	Stomach	Surgical operation+chemotherapy	Death
11 [18]	2015	18	Melena	Stomach	Chemotherapy	Live
12 [19]	2019	30	Melena	Duodenum	Chemotherapy	Death
13 [20]	2020	17	Melena	Duodenum	Chemotherapy	Live
14 [21]	2021	32	Melena	Jejunum	Surgical operation+chemotherapy	Death
15 [22]	2021	33	Melena	Intestinum tenue	Surgical operation+chemotherapy	Death
16 [23]	2021	40	Melena	Duodenum	Chemotherapy	Live
17	2022	28	Melena	Intestinum tenue	Surgical operation+chemotherapy	Live

Death refers to death during hospitalization. The 17th case is the patient of this article

The determination of serum tumor markers HCG and AFP may be helpful in the diagnosis of choriocarcinoma because they are elevated in about 80% of cases. The serum concentration of HCG can also be used to monitor the response to treatment. According to the international cooperative organization for germ cell cancer, HCG higher than 50,000miu/ml indicates poor prognosis. However, in this paper, the monitoring of HCG level after radical resection of testicular cancer is ignored, which leads to multiple metastasis and poor prognosis. In addition, as a transcription factor, GATA3 is another immune tumor marker sensitive to choriocarcinoma [24].

The imaging of testicular choriocarcinoma lacks characteristic changes that can be distinguished from other types of germ cell tumors. It is difficult to diagnose choriocarcinoma first. Most of the specimens obtained during surgical resection are confirmed by pathology. The typical histopathological feature of metastatic choriocarcinoma is the coexistence of cytotrophoblast and syncytiotrophoblast cells without mesenchymal cells, which is different from other germ cell tumors with only scattered syncytiotrophoblast cells.

The treatment of testicular choriocarcinoma depends on the stage of the disease. Radical orchiectomy and dissection of affected lymph nodes are the treatment of early diseases. The treatment of bleeding caused by gastrointestinal metastasis of choriocarcinoma is similar to that of other gastrointestinal bleeding, including endoscopic intervention, embolization, or surgical resection. Abdelkader et al. reported a case of bleeding from duodenal choriocarcinoma. The exudation point was ablated by an endoscopic adrenalin injection and argon plasma coagulation system to finally stop bleeding [18]. Bain et al. [13] reported a case of bleeding treated by angiography and embolization. Iglesias et al. [25] used surgical hemostasis after endoscopic injection of adrenalin and argon plasma coagulation system ablation failure. In our literature review (Table 1), we found that 6 patients received surgical treatment, but only 1 patient survived (survival rate 16.7%), while 5 patients survived (50%) in 10 patients who received chemotherapy only without surgery. This result may be related to the fact that patients undergoing surgery generally have a large amount of bleeding and serious condition, and surgical intervention must be taken. And in this case of our paper, because there was no bleeding focus under endoscopy and selective angiography, the patient finally stopped bleeding by surgical means.

Chemotherapy consolidation is usually required after bleeding stops which is also consistent with the results of our review of case reports in recent 20 years. For metastatic choriocarcinoma, the platinum therapy is recommended as the first-line chemotherapy, but unfortunately, choriocarcinoma is not so sensitive to chemotherapy. Most patients’ tumors progress so rapidly that they do not respond to the standard chemotherapy regimen of three to four cycles of BEP (bleomycin, etoposide, and cisplatin) [26]. In recurrent cases, salvage chemotherapy with vincristine and ifosfamide may help to reduce the tumor burden, but these patients may finally have to choose palliative treatment. Simple testicular choriocarcinoma usually has a poor prognosis, with a 5-year survival rate of less than 80% [4]. In some reports, the long-term survival rate is even lower [27, 28], while mixed choriocarcinoma is slightly better. In this paper, the patient was treated with paclitaxel, ifosfamide, and cisplatin.

Testicular choriocarcinoma is a rare tumor with strong invasiveness and rapid growth in young men. It mainly metastasizes to the lung, liver, and brain. Reports of metastasis to the gastrointestinal tract are rare, which makes it easy to ignore the existence of the disease in the clinical diagnosis and treatment of gastrointestinal bleeding. Therefore, we recommend a more detailed inquiry into medical history and systematic examination. It is very important for us to correctly distinguish and diagnose the etiology of gastrointestinal bleeding. The lack of accurate physical examination and laboratory examination will lead to waste of patients’ diagnosis and treatment time, the increase of mortality, the extension of hospital stay, and the increase of patients’ medical expenses. Therefore, for any young male patient, the most basic reproductive system examination is very important. Among the patients in this article, the reproductive system examination was omitted during the patient’s repeated hospitalization outside the hospital, and it was not carried out when he was admitted to our hospital. In addition, the detection of HCG was also omitted. Although the final surgical pathology guided us to diagnose testicular choriocarcinoma, during the process, the patient once had hemorrhagic shock, which may have been life-threatening, and we do not know how many patients died because their doctors missed this article.

## Conclusion

Testicular choriocarcinoma is a rare malignant tumor with early metastasis. Although it is extremely rare, gastrointestinal metastasis of choriocarcinoma should be a part of the differential diagnosis of upper gastrointestinal bleeding in young male patients. The earlier we diagnose the disease, the greater the opportunity for us to start treatment in the golden age, and the mortality, hospital stay, and treatment cost will be greatly reduced.

## Data Availability

The data that support the findings of this study are available on reasonable request from the corresponding author. The data are not publicly available due to privacy or ethical restrictions.

## Abbreviations

CT: Computed tomography
AFP: Alpha fetoprotein
HCG: Human chorionic gonadotropin
CK: Cytokeratin
CD: Cluster of differentiation
PLAP: Placental alkaline phosphatase
CDX-2: Caudal-type homebox-gene transcription factor-2
SATB: Special AT rich sequence-binding protein
TTF-1: Thyroid transcription factor-1
OCT3/4: Octamer binding transcription factor 3/4
GATA3: GATA-binding protein 3
